# Multi-State Proteins: Approach Allowing Experimental Determination of the Formation Order of Structure Elements in the Green Fluorescent Protein

**DOI:** 10.1371/journal.pone.0048604

**Published:** 2012-11-14

**Authors:** Tatiana N. Melnik, Tatiana V. Povarnitsyna, Anatoly S. Glukhov, Bogdan S. Melnik

**Affiliations:** Institute of Protein Research, RAS, Pushchino, Moscow Region, Russia; University of Maryland School of Medicine, United States of America

## Abstract

The most complex problem in studying multi-state protein folding is the determination of the sequence of formation of protein intermediate states. A far more complex issue is to determine at what stages of protein folding its various parts (secondary structure elements) develop. The structure and properties of different intermediate states depend in particular on these parts. An experimental approach, named μ-analysis, which allows understanding the order of formation of structural elements upon folding of a multi-state protein was used in this study. In this approach the same elements of the protein secondary structure are “tested” by substitutions of single hydrophobic amino acids and by incorporation of cysteine bridges. Single substitutions of hydrophobic amino acids contribute to yielding information on the late stages of protein folding while incorporation of ss-bridges allows obtaining data on the initial stages of folding. As a result of such an μ-analysis, we have determined the order of formation of beta-hairpins upon folding of the green fluorescent protein.

## Introduction

Fundamental studies of denaturation and renaturation of small two-state proteins have allowed us to develop experimental approaches for obtaining information on the effect of individual amino acid residues on the protein energy landscape (see, e.g. [1], [2]). One of such approaches is the phi-analysis using which we can determine amino acid residues included in the so-called “folding nucleus” (i.e. the structured part of the “transition state”). This approach consists in measuring and analyzing the folding and unfolding rates of the wild-type protein and its mutant forms (with single amino acid substitutions) at various concentrations of the denaturant [3]–[5]. At the same time, denaturation and renaturation of comparatively large proteins (more than 20 kDa) occurring according to the multistage mechanism (i.e. with accumulation of more than one intermediate state) have been studied quite insufficiently. Experimental studies of complex proteins with stable intermediate states in their folding are rather complicated. The main difficulty lies in the treatment and interpretation of intricate kinetic data, inasmuch as the more the number of different states in protein, the more difficult it is to differentiate them and to appreciate their formation order and mutual influence.

In this study we have investigated the green fluorescent protein (to be more exact, analogous GFP-cycle3 close to the naturally occurring protein) upon folding of which at least two intermediate states are formed [6]–[9]. Having analyzed mutant forms of this protein, we tried to understand the formation order of its structure elements. GFP-cycle3 seems to be the most appropriate protein for such studies. First, the chromophore formation and fluorescence of mutant forms of this protein evidences unambiguously that the mutation did not disturb the general protein structure. Second, the structure of GFP-cycle3 represents a β-can and allows designing almost at any place not only single amino acid substitutions but also ss-bridges between neighboring beta-strands. We used two different types of mutations in our study: single substitutions of hydrophobic amino acids with a great number of contacts and incorporation of ss-bridges. Our microcalorimetric analyses of the mutant forms of this protein when its melting is a non-equilibrium process allowed us not only calculate the rate constants of the GFP-cycle3 unfolding but also obtain data on the effect of these mutations on the entropic and enthalpic components of the protein energy barriers. In turn, this permitted us to establish the formation order of beta-hairpins upon the GFP-cycle3 folding.

## Results

### Choice of Mutations

Hydrophobic interactions and protein topology affect largely the stability of protein and the formation of its intermediate states. Naturally there are many other important interactions (hydrogen bonds, charge-charge interactions), but the contribution of hydrophobic amino acids and protein topology are yet the most critical. Provided contacts are essential, it would be logical to investigate how substitutions of hydrophobic amino acids with a large amount of contacts affect the protein. If topology is vital, it would be logical to “slightly” influence it, for example, by introducing ss-bridges in secondary structure elements and analyzing their effect on the protein. And in our opinion, it would be far more logical to carry out a symmetrical study of the influence of the both types of mutation on stability, folding rate and formation of intermediate states of multi-state proteins. Nearly such considerations were applied to choose mutations in GFP*-*cycle3. However there was also another circumstance that inspired us to make this research. Having analyzed literature data on the effect of incorporated ss-bridges on the proteins [10]–[16] and having studied the folding of several multi-state proteins [12], [17]–[20], we concluded that the introduction of an ss-bridge affects mainly early intermediate stages upon protein folding, whereas single substitutions of hydrophobic amino acids affect late intermediate stages. Therefore a systematic investigation of the two types of mutations would yield more information on different stages of formation of the structure of multi-state proteins.

**Figure 1:**
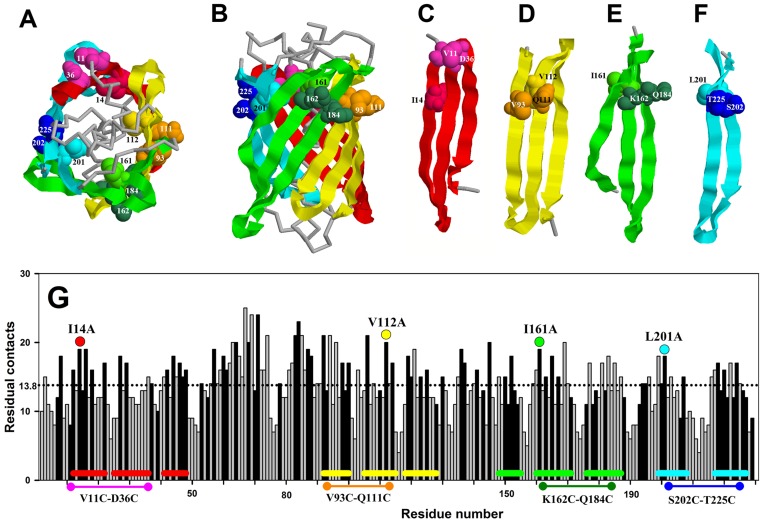
Choice of mutations in GFP-cycle3. End view (A) and side view (B) of the voluminous model of the GFP-can. Four structure elements are shown individually: (C–E) three β-sheets and (F) β-hairpin. Balls denote amino acids substituted for alanine (I14, V112, I161, L201) and pairs of amino acids substituted for cysteines (V11 and D36, V93 and Q111, K162 and Q184, S202 and T225). Plot G shows the amount of residue-residue contacts of each amino acid residue (a contact distance is 6 Å. Black columns denote hydrophobic amino acids. Coloured lines at the bottom of plot G denote the position of β-strands in structure elements C–F. Coloured circles in plot G show columns referring to amino acids substituted for alanine. Coloured circles connected by a line at the bottom of the plot mark pairs of amino acids substituted for cysteines.

The above considerations of how different mutations would affect the protein severely restrict the choice of mutations. Let us try to comply with this by choosing individual structure elements in GFP*-*cycle3 that are most likely formed at different stages of the protein folding. Besides we will also calculate the amount of contacts in every amino acid within GFP*-*cycle3. Then in every structure element we will choose a hydrophobic acid with a great number of contacts and next to it a pair of adjacent amino acids (on the protein surface) that can form an ss-bridge when they are substituted for cysteines. Figure 1 shows a three-dimensional model of GFP*-*cycle3 (A and B), individual structure elements in which amino acids were substituted (C–F), and the plot demonstrating the amount of residue-residue contacts in every amino acid (G). The substituted amino acids are shown in three-dimensional models and in Fig. 1G. The hydrophobic amino acids chosen for substitutions are immersed in GFP*-*cycle3 (Fig. 1A) and have a great number of residue-residue contacts (Fig. 1G). In protein chain regions of 150–190 a.a. and 200–230 a.a., amino acids I161 and L201, having the greatest amount of contacts among the hydrophobic amino acids in these regions, were chosen to be substituted by alanine. In the protein region of 90–130 a.a., amino acid V112 was chosen to be substituted. Amino acids Y92 and Y106 also having a great number of contacts are not fairly suitable for substitution because they are aromatic amino acids and their replacement could affect spectral properties of the protein in further studies. The protein region of 10–50 a.a. contains two equal “candidates” for substitution – I14 and V16. Finally we have chosen I14 because this amino acid is located closer to the ends of the β-hairpin (Fig. 1C). Pairs of amino acids substituted for cysteines were chosen according to three criteria. First, the mutual positions and orientation of these amino acids should be such that an ss-bridge could be formed after their substitution. Second, they should be located as close as possible to the amino acids that will be replaced by alanine. Third, the ss-bridge should “cross-link” the ends of the β-hairpin. The above-stated is demonstrated in Figure 1. So GFP*-*cycle3 proteins with mutations I14A, V112A, I161A, L201A, V11C and D36C, V93C and Q111C, K162C and Q184C, S202C and T225C were designed and prepared.

### Heat Denaturation of GFP-cycle3

When we began this research, we had a very narrow set of experimental methods that could be used in studying GFP*-*cycle3. After studying literature data and conducting preliminary experiments [6], [21], [22], we understood that because of the strong aggregation of GFP*-*cycle3 upon folding it was possible to measure reliably only constants of unfolding rates of this protein. At the same time even if we succeed to get constants of unfolding rates at all stages of GFP*-*cycle3 unfolding with optical methods, this would not allow understanding the sequence of unfolding of different states of this protein. But preliminary microcalorimetric experiments on protein melting demonstrated that the heat denaturation of GFP*-*cycle3 is non-equilibrium: the position and shape of the melting curve depend on the heating rate. As a result, such thermodynamic functions of structure stabilization as entropy and Gibbs free energy cannot be obtained from microcalorimetric curves. Nevertheless, in these cases the shape of melting curves can provide very useful information about kinetic parameters of denaturation [23]–[28]. In addition, it is also possible to accurately determine the sequence of different stages of protein melting, i.e. it would be possible to define the rate of the first stage of protein melting and the rate of the following stage.

**Figure 2:**
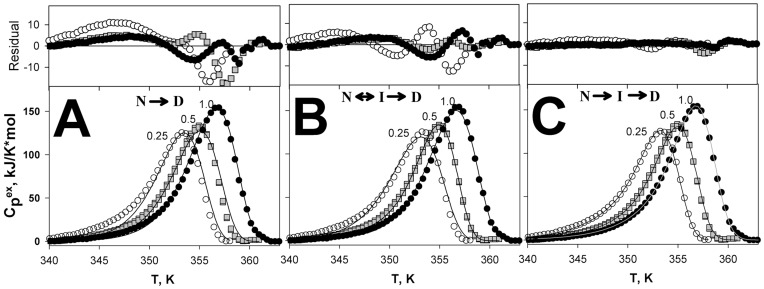
GFP-cycle3 melting and choice of irreversible denaturation models. Dependences of excess molar heat capacity versus temperature for GFP-cycle3 measured at three scanning rates (1.0, 0.5, 0.25 K/min). Symbols show experimental data and lines show data calculated with the use of one-stage model (A), the Lumry-Eyring model with the fast equilibrating first step (B) and irreversible models involving two consecutive irreversible steps of denaturation (C). Residual plot for fitting is shown in the upper panels.

In our previous paper differential scanning microcalorimetry was used to study in detail non-equilibrium melting of GFP-cycle3 and to characterize its transition states [9]. Therein we also defined the model of denaturation and sequence of unfolding stages of this protein. Figure 2 shows melting curves of GFP-cycle3 and approximation of the curves over three models of denaturation.

The model of one-stage irreversible denaturation is as follows:
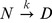
(1)


The Lumry-Eyring model with the fast equilibrating first step, in which the rate of equilibrium establishing (*K*) for the first step is high in comparison with that (*k*) of the second step:
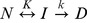
(2)


Model involving two consecutive irreversible steps of denaturation:
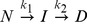
(3)(*N, I, D* are different states of the protein; k is constants of protein unfolding rates).

Fig. 2 shows that only the third model (two consecutive irreversible steps of denaturation) permits good approximation of the experimental melting curves obtained at three various heating rates. In particular, it is seen from Fig. 2 that if one individual melting peak (for example, at the rate of 0.5 K/min in Fig. 2 B and C) can be described satisfactorily using different models, only one model can describe well all three melting peaks. This is explained by that the shift of melting curves at different heating rates is an additional parameter allowing us to choose accurately the denaturation model. The details of calculations and the choice of models are described in our previous paper [9] where we used an additional criterium to differentiate non-equilibrium one-stage melting from non-equilibrium multi-stage melting [23]. It should be noted that when approximating curves of non-equilibrium melting we determine not separate values of rate constants but parameters controlling the total dependence of rate constants on temperature (ln(*k*) vs (1/*T*), see Materials and Methods). The sequence of denaturation stages is unambiguously determined from approximation of curves of non-equilibrium melting. Upon mutual substitution of rate constants in equation 9 (if *k_1_* is substituted by *k_2_* and vice versa) the result of calculations of the excess heat capacity will be changed. That is why their sequence is of importance and is determined unambiguously. Moreover, the dependence of the logarithm of rate constants of folding/unfolding versus temperature (to be more exact, versus reverse temperature) allows differentiating the effect of mutation on the entropy and enthalpy components of energy barriers. The values of logarithms of folding/unfolding rate constants are proportional to the free energy value of the activated state of protein (both the enthalpic and entropic components) and the slope of these dependencies is related only to the enthalpic component of free energy.

**Figure 3:**
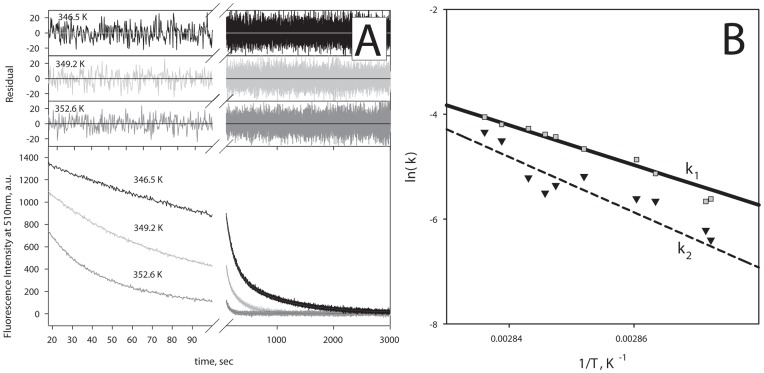
Rate constants of GFP-cycle3 unfolding obtained with the fluorescence method is well compatible with the same obtained with the calorimetry method. (**A**) Typical kinetic unfolding curves of GFP-cycle3 at pH 6.2 caused by a temperature jump from 293.2 K to 346.5 K, 349.2 K and 352.6 K, accordingly. The curves (noisy lines) were well fitted to two exponentials (continuous lines). Residual plot for fitting is shown in the upper panel. (**B**) Dependences of the logarithm of rate constants (*k_1_* and *k_2_*) of unfolding of GFP-cycle3 versus reverse temperature. Rate constants obtained with the fluorescence method are shown by symbols. Rate constants obtained from calorimetric experiments are shown by lines.

Besides inasmuch as such a calorimetric approach to determination of kinetic parameters of denaturation is not frequently used, we conducted a direct kinetic experiment and measured the thermal unfolding of GFP-cycle3 using the fluorescence method. Fig. 3A shows kinetic curves measured with this method. The kinetics was approximated by exponential equations which allowed us to calculate unfolding rate constants. From Fig. 3B showing the calorimetric and fluorescence data (rate constants of thermal unfolding) it is seen that the dependence of logarithms of rate constants of GFP-cycle3 unfolding obtained with the fluorescence method is well compatible with the same dependence obtained with the calorimetry method. Though we cannot get any information on the model of unfolding or the sequence of unfolding stages from the fluorescence data, the coincidence with the calorimetric data supports the reliability of the latter. Therefore it may be concluded unambiguously that the melting of GFP-cycle3 can be described with a two-stage non-equilibrium model.

As a result of the detailed investigation of heat denaturation of GFP-cycle3 with the microcalorimetry method, we have obtained a “tool” for studying the influence of mutations of amino acid residues on different stages of GFP-cycle3 unfolding.

### Microcalorimetric Study of Heat Denaturation of the GFP-cycle3 Mutants

**Figure 4:**
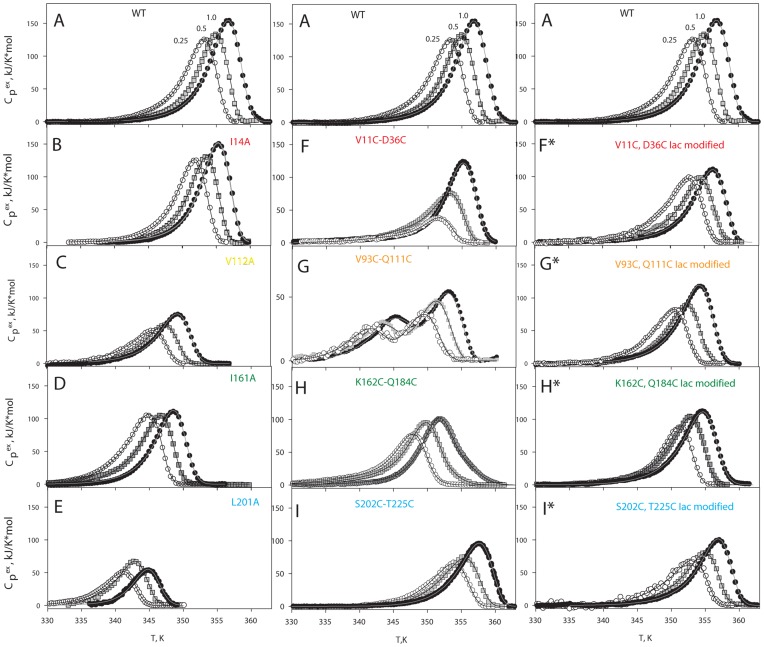
Melting curves of GFP-cycle3 (WT) and its mutants measured at three heating rates. Every top plate (A) shows melting curves of WT for comparison. B–E, Melting curves of mutant proteins with single substitutions of hydrophobic amino acids for alanine. F–I, Melting curves of mutant proteins with double substitutions for cysteines and oxidized ss-bridges. F*–I*, melting curves of mutant proteins with double substitutions for cysteines modified with iodacetamide (the ss-bridge is not formed). Symbols show experimental data (○ 0.25, ▪ 0.5, • 1.0 K/min), and lines show the best fit of the experimental data using equation 9 and 10.

Figure 4 shows melting curves for both GFP-cycle3 and its mutants with single substitutions of hydrophobic amino acids and incorporation of ss-bridges. Below GFP-cycle3 will be denoted as WT (wild type). We have also performed melting of mutant proteins containing cysteines modified with iodacetamide to clarify how much cysteines themselves (without the cross-linking ss-bridges) affected the protein. Figure 4 demonstrates that at three heating rates (1.0, 0.5 and 0.25 K/min) the melting curves of each protein vary from each other in the temperature of maxima (they are shifted along the temperature axis) and in their shape. Such behavior is typical of non-equilibrium melting of protein. Consequently, the melting of all proteins studied is non-equilibrium. The paper early chapter and in our previous paper [9] we described in detail the investigation of GFP-cycle3 melting at different heating rates, different pH and different concentrations of the protein. It was shown that the melting of GFP-cycle3 can be described by the equation of two-stage irreversible denaturation [9], i.e. protein denaturation occurring in accord with the model involving two consecutive irreversible steps of denaturation (eq. 3). The correctness of the chosen model is also supported by investigations of mutant proteins. For example, since the Lumry-Eyring model (eq. 2) suggests the existence of an equilibrium stage of melting, this model cannot describe the protein melting with the substitution V93C-Q111C (Fig. 4 G). The both maxima on the melting curve of this protein are dependent on the heating rate which is a direct evidence of the existence of two non-equilibrium stages of melting.

**Figure 5:**
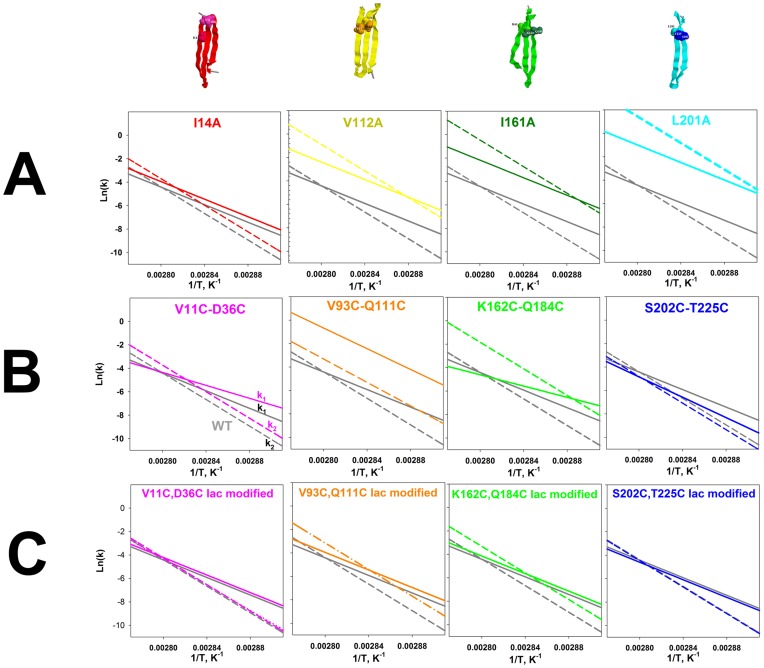
Arrhenius plots of GFP*-*cycle3 and its mutant forms. Dependencies of logarithms of rate constants of the first (*k*_1_) (solid line in the figures) and second (*k*_2_) (dashed line) stages of GFP*-*cycle3 heat denaturation versus inverse temperature calculated from melting curves of GFP*-*cycle3 (gray lines) and its mutant forms (colored lines). **A**, the plots are related to singular substitutions of hydrophobic amino acids. **B**, the plots are related to double substitutions for cysteines with an ss-bridge between them. **C**, the plots are related to double substitutions for cysteines modified with iodoacetamide (an ss-bridge is not formed).

Having performed concurrent fitting of the melting curves at three heating rates (eq. 9, Materials and Methods,) it is possible to calculate enthalpy (*ΔH*), activation energy (*ΔE^#^*) and parameter T* for two stages of denaturation of each protein [9]. All the calculated parameters are listed in Table 1. They allow plotting the dependency of the logarithm of rates of the first (*k_1_*) and second (*k_2_*) stages of heat denaturation versus reverse temperature (the Arrhenius plot). Figure 5 shows such dependencies for all proteins studied. In each plot, grey lines refer to the wild-type protein (WT) and colored lines to a corresponding mutant protein. This figure is the basic experimental result, because it permits performing a comparative analysis of the effect of single mutations and ss-bridges on protein. As an example let us analyze the Arrhenius plot for a mutant protein with an ss-bridge between amino acids 11 and 36 (V11C−D36C in Fig. 5). It is clearly seen in the figure that this mutation affected the slope of the Arrhenius plot for rate constant *k_1_* and had no effect on the slope of the Arrhenius plot for rate constant *k_2_*. The shift of the Arrhenius plot shows that the mutation changed only the entropic component of the transition state and its changed slope indicates that the mutation also affected the enthalpic component of the transition state. Figure 5 demonstrates that single substitutions of amino acids and replacements of amino acids by cysteines modified with iodacetamide (without formation of ss-bridges) influence only the shift of the Arrhenius plot without changing its slope. This means that such mutations affect only the entropic component of energy barriers. In contrast, in all cases the formation of an ss-bridge changed the slope of the Arrhenius plot, i.e. the enthalpic component of energy barriers. Table 1 demonstrates this numerically. If parameter *ΔE^#^* (activation energy) for the mutant protein is the same as that for the WT protein, the mutation affects only the entropic component of the energy barrier.

**Table 1:** Combined fitting parameters of heat denaturation curves of GFP*-*cycle3 and its mutants using a model involving two consecutive irreversible steps of denaturation (eq. 9, 10).

	Scan rate, K/min	Δ*H*_1_±5, kJ/mol	Δ*H*_2_±6, kJ/mol	Δ*E*_1_^#^±4, kJ/mol	Δ*E*_2_^#^±2, kJ/mol	*T*_1_*±0.5, K	*T*_2_*±0.2, K
GFP*-*cycle3 (WT)	1	357	611				
	0.5	269	542	311	471	373.4	367.2
	0.25	278	534				
I14A	1	244	645				
	0.5	199	567	311	471	371.1	365.7
	0.25	201	568				
V112A	1	283	220				
	0.5	211	190	311	471	365.5	359.2
	0.25	105	169				
I161A	1	298	396				
	0.5	357	337	311	471	364.7	358.2
	0.25	372	359				
L201A	1	379	262				
	0.5	191	226	311	471	360.5	353.9
	0.25	325	260				
V11C-D36C	1	202	582				
	0.5	236	351	230	471	378.4	365.7
	0.25	97	187				
V93C-Q111C	1	222	348				
	0.5	189	294	367	415	359.1	365.8
	0.25	163	231				
K162C-Q184C	1	242	555				
	0.5	343	401	199	472	383.5	361.3
	0.25	264	324				
S202C-T225C	1	364	275				
	0.5	350	177	361	471	371.9	368.3
	0.25	273	188				
V11C, D36C	1	266	453				
Iac modified	0.5	236	410	311	471	372.1	367.0
	0.25	270	434				
V93C, Q111C	1	381	401				
Iac modified	0.5	260	316	311	471	371.1	364.4
	0.25	275	288				
K162C, Q184C	1	425	344				
Iac modified	0.5	332	354	311	471	371.8	364.8
	0.25	178	361				
S202C, T225C	1	290	378				
Iac modified	0.5	289	350	311	471	373.5	367.5
	0.25	199	215				

Fitting errors are given. See experimental errors in Materials and methods.

## Discussion

### Some Speculative Models that Facilitate the Explanation of the Effect of Two Types of Mutations on Folding/Unfolding of a Multi-state Protein

The models and considerations described here appeared upon receiving the experimental data and their subsequent analysis. That is why on the one hand, the models contribute to understanding the results of the experiments, and on the other hand, the results of the experiments allowed us to design models described below. In particular, after analysis of the effect of mutations on the entropy and enthalpy components of energy barriers (see the next section) it can be supposed that the introduced ss-bridges affect the transition states of the protein. So this effect on the energy landscape of the protein is taken into account in the below schemes of unfolding.

As concerns hydrophobic amino acid residues with a large number of contacts, in our opinion, they will influence primarily the stable states of the protein (native *N* and intermediate *I*). Such a proposal can be made taking into account the following factors. First, by definition, the intermediate state is the most unstable one upon protein folding. So many researchers believe that the polypeptide chain in this state should be packed more or less “correctly” (because of which the protein “loses” the entropy component of the free energy), but at the same time there are no tight contacts of amino acid residues (because of which the protein “loses” also the enthalpy component of the free energy). Second, numerous investigations of the transition state with the phi-analysis demonstrated that the protein has many amino acid residues with a low value of phi (<0.5) and only few residues with phi >0.5 [20], [29]–[32]. This shows that the most part of the contacts of amino acid residues realized in the native state are not realized in the transition state. What does this mean? Let us examine an amino acid with a single contact. In this case it is impossible to predict whether such a contact is essential (phi>0.5) or not essential (phi<0.5) for the formation of a transition state. Can we say anything about a residue with multiple contacts (it contacts with a large number of other amino acids)? Naturally we can, because it is known from the phi-analysis that the major part of amino acid residues contacting with the given amino acid have a low value of phi, that it they cannot form native contacts in the transition state. So, if a residue has more contacts with other amino acid residues, the higher is the probability that this residue would not form all of its contacts in the transition state. Namely this demonstrates that such a residue is not of great importance for the transition state but is critical for stable (native or intermediate) states.

Let us try to predict what experimental results may be expected when studying both substitutions of hydrophobic amino acid residues with a great number of contacts and incorporation of ss-bridges in a multi-state protein.

**Figure 6:**
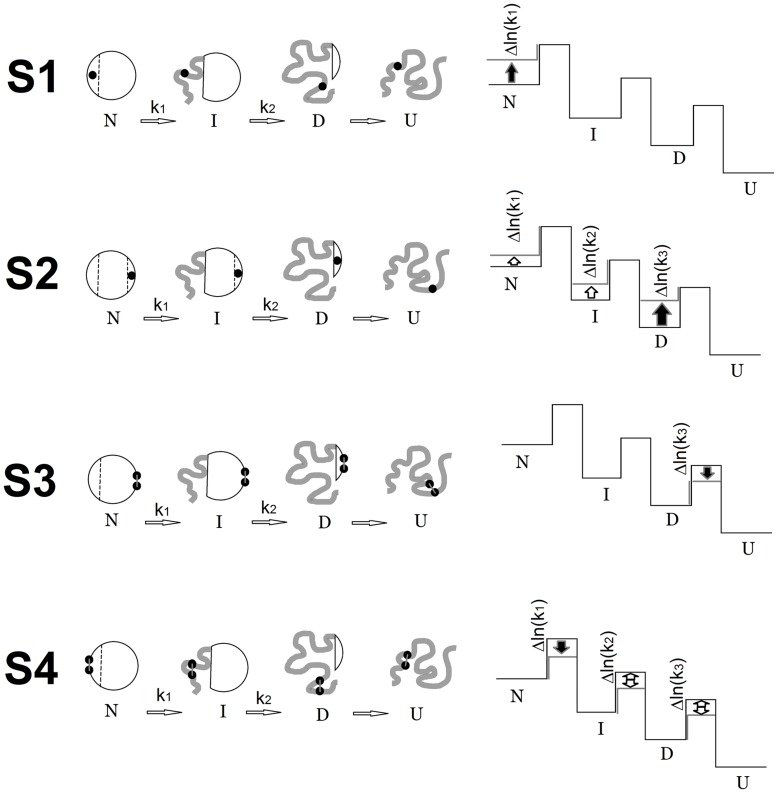
What changes in energy levels of a multi-state protein could be expected if we make single mutation or ss-bridge incorporation. Schematic representation of the sequence of states at unfolding of GFP (on the left) and relative arrangement of energetic levels (on the right) of this protein. *N*, *I*, *D*, and *U* are native, intermediate, denatured and unfolded states of the protein, respectively. Substituted amino acid residues (mutations) are shown with black circles. **S1**, the substituted amino acid residue is contained only in the structure of native state. Therefore destabilization of the native state should lead to acceleration of only the first stage of unfolding (*k*1). **S2**, the substituted amino acid residue is included in the structure of the very last state (*D*) hence it should affect the stability of all states and also the rates of all unfolding stages. **S3**, the ss-bridge (two black circles in the figure) in the structured part of state *D* would influence only the very last stage of unfolding because it does not affect the mobility of states *N* and *I*. It is assumed that we have introduced an ideal ss-bridge that has not impaired the protein internal packing. **S4**, the ss-bridge in the structured part of state *N* would influence all the stages of unfolding because it “forces” the protein region that should unfold during the first stage to be compact. There is an intentional inaccuracy in the represented energetic schemes. In fact, if an intermediate state *I* (for example in S2) is destabilized by a mutation, the free energy of the transition state between *N* and *I* should change at least by the same value. In this figure, the energetic schemes are represented in assumption that every unfolding stage occurs independently of all the other processes. Since only the unfolding rates are analyzed here, such a simplification of the energetic schemes is quite reasonable and provides an easy way to compare these rates with each other and to relate theoretical considerations with the experimental data.

Figure 6 shows a scheme of the order of states upon unfolding of a protein with two intermediate states (left) and the mutual positions of energy levels of this protein (right). Below we will discuss the unfolding of GFP-cycle3. First let us analyze what changes in energy levels of a multi-state protein could be expected if we make single substitutions of hydrophobic amino acid residues with multiple contacts. As explained above, such mutations should largely affect tightly packed states. Suppose we have substituted the amino acid in the structure element which is formed at the latest stage of folding (Fig. 6, S1). It is clear that such a substitution destabilizes only the native state and has no effect on the other states because even after the first stage of unfolding this amino acid is in the “distorted” unstructured region of the protein. It is seen from the energy scheme (Fig. 6, S1) that this mutation should affect only the rate of the native state unfolding (*k_1_*). In contrast, if the substituted residue stabilizes the structure element that is the latest to unfold (and the first to fold), such a mutation would have an effect on all stable states of the protein as demonstrated in Fig. 6, S2. As each of the following states of the protein (upon its folding) includes the preceding one, this substitution would cause a change in all the unfolding rates (Fig. 6 S2).

Therefore the later the region with the mutation is structured (i.e. nearer to the native state) the lesser amount of states will be “destroyed” as a result of this mutation (Fig. 6 S1). On the contrary, the earlier the region with the mutation is structured (i.e. nearer to the unfolded state) the greater amount of states will be affected by this mutation (Fig. 6 S2).

Figure 6 demonstrates how the protein unfolding is changed after the ss-bridge incorporation when the latter cross-links the small structure element that unfolds the last (Fig. 6 S3) or the first (Fig. 6 S4). The incorporation of the ss-bridge should contrariwise affect various stages of protein unfolding as compared to single mutations. That means that if we “ss-bridged” the structure that unfolds the last (folds the first), we will affect only one stage of unfolding/folding (Fig. 6 S3). And in case we “cross-linked” the structure element that unfolds the first (folds the last), then evidently such a mutation would change the whole pathway of unfolding/folding (Fig. 6 S4) because it would “force” the unstable structure element to retain its compact conformation in all stages of unfolding. It should be underlined that in figure 6 it is difficult to show minor details of mutual influence of different energy levels of the protein. The exclusive aim is to schematically demonstrate distinctive features of the effect of such mutations on the energy landscape of a multistate protein.

### The Analysis of Action of Two Types of Mutations on a Multi-state Protein

**Figure 7:**
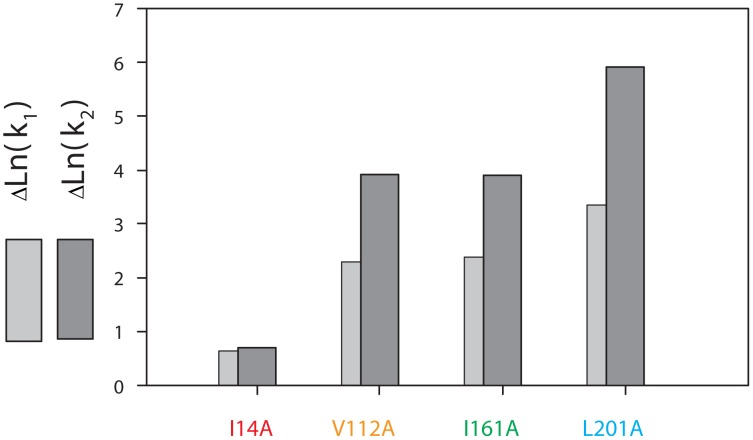
Effect of single amino acids substitutions on GFP*-*cycle3 unfolding rate constants. Change of logarithms of unfolding rate constants for GFP*-*cycle3 mutant proteins with single amino acids substitutions as compared to the WT (GFP*-*cycle3) protein: ΔLn(*k*_1,2_) = ΔLn(*k*_1,2_)_mut_− ΔLn(*k*_1,2_)_WT_.

The analysis of action of various mutations on the folding/unfolding of a multi-stage protein is not complicated although it requires some logical plotting. Figure 7 shows changes in the logarithms of two rate constants (*k_1_* and *k_2_*) for mutant proteins with single amino acid substitutions. In effect, this is a numerical expression of the shift of colored lines relative to grey ones in Figure 5. The higher the column in Figure 7, the more prominent the effect of mutation on the corresponding rate constant. It is seen that mutation I14A had an approximately the same effect on the both rate constants (*k_1_* and *k_2_*) and this effect is quite weak as compared to the other single amino acid mutations. However it should be reminded that all amino acids chosen for single substitutions are amino acids with the greatest number of contacts (Fig. 1G). That is each of the substitutions should strongly affect the stability of the intermediate state in which its contacts are formed. Previously we have discussed in detail such an effect (see Fig. 6 and the discussion in the text). The fact that the I14A substitution affected the initial stages of unfolding much weaker than the other mutations indicates that the protein region at amino acid I14 unfolds later than in the region of other single substitutions. The effect of mutation I14A is similar to that demonstrated in Fig. 6 S2, while the effect of mutations V112, I161 and L201 is similar to that shown in Fig. 6 S1 the difference being that mutations V112, I161 and L201 caused changes in two stages of unfolding and not only the very first one as in Fig. 6 S1.

From the analysis of the effect of single mutations on GFP-cycle3 it can be concluded that *upon folding of GFP-*cycle3 *the part of the protein chain in region I14 unfolds later than in regions V112, I161 and L201.*

Let us focus our attention on the Arrhenius plots for mutant proteins containing cysteines modified with iodacetamide, i.e. when ss-bridges are not formed (Fig. 5C). It is seen that for such proteins the lines in the plots (ln(*k*) vs 1/*T*) either are compatible with the lines for WT or vary from them by a slight shift. This means that modified cysteines did not affect the slopes of these dependencies. At the same time Fig. 5B demonstrates that oxidized (cross-linked) ss-bonds changed the slopes of the dependencies (ln(*k*) vs 1/*T*). First we will analyze only the change in the slopes because a comparison of the plots for mutant proteins with “cross-linked” and “non-cross-linked” ss-bridges (Fig. 5B) shows that just the slope of the dependencies (ln(*k*) vs 1/*T*) is determined by the formation of an ss-bridge. It is seen that all ss-bridges affected the slope of the ln(*k_1_*) dependency on reverse temperature and only mutation V93C-Q111C had an effect on both the slope of the ln(*k_1_*) dependency and that of ln(*k_2_*). As has been stated above (see Fig. 6 S3, S4), the earlier the structure element with an incorporated ss-bridge unfolds, the greater is the amount of states influenced by such a mutation. *Consequently, it can be concluded that the earliest to unfold is the beta-hairpin cross-linked by an ss-bridge between V93 and Q111* (the “yellow” one in Fig. 1), because this mutation changed both rate constants *k_1_* and *k_2_* whereas the other three mutations changed only *k_1_*. As a result, even a visual analysis of the experimental data permits us to predict in the first approximation the order of unfolding of structure elements of GFP-cycle3. In Figure 1 different structure elements of GFP-cycle3 are distinguished using four colours. In accord with these colours, the order of unfolding of protein structure elements can be written as follows.

*N* → (unfolded yellow) → (unfolded green and blue) → (unfolded red) → *U.*

**Figure 8:**
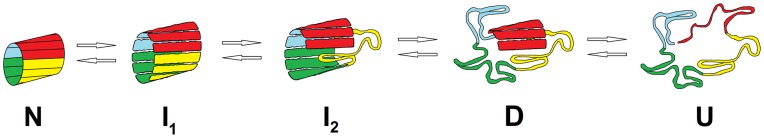
GFP*-*cycle3 structure formation. Sequence of formation/distortion of secondary structure elements of GFP*-*cycle3 based on the results of multimutational analysis. *N, I_1_, I_2_, D*, and *U* are native, two intermediate, denatured and unfolded states of the protein, respectively. At the first stage of unfolding (*N*→*I_1_*) the packing of amino acids on the protein surface is impaired, but the packing of the hydrophobic nucleus as well as the entire packing of the protein chain are not distorted. During the following stage (I*_1_*→*I_2_*) the structure of β-strands (shown in yellow color) is changed (in the region of amino acids 90–130). After that the protein unfolds almost completely (*I_2_*→*D*), excluding three β-strands in the region of amino acids 10–50 which at “mild” denaturation remain structured in the unfolding state. The last stage (*D*→*U*), when the total protein acquires a coil-like conformation, is probable only under the action of strong denaturants.

As mentioned above, the shifting of the ln(*k*) dependency on reverse temperature at a stable slope (the same activation energy value for GFP-cycle3 and its mutant) means that the mutation affected only the entropy component of the energy barrier. The effect on the slope of (ln(*k*) vs 1/*T*), imply the effect on the enthalpic component of the energy barrier. Therefore from Fig. 5 it can be concluded that amazingly all ss-bridges had an effect on the enthalpic component of the first (upon unfolding) energy barrier. It appears that independent of its position on the surface of GFP-cycle3 any ss-bridge affects some process that is the first to take place upon unfolding. In other words, upon unfolding the first is some change in the structure of GFP-cycle3 that involves the total protein. This can hardly be a change in the hydrophobic nucleus packing. As we have seen, the substitutions of hydrophobic amino acid residues had different effects on the first stage of unfolding (Fig. 5A). Most likely, this is the process of the final packing (upon folding) and mutual orientation of amino acid residues on the protein surface. It is just such a process that can take place the last upon folding and the first upon unfolding over the entire protein surface. Why do changes occur specifically in the enthalpic component of the energy barrier? It can be suggested that primarily ss-bridges should affect the transition states of the protein. The ss-bridges “strengthen” amino acid interactions around themselves (as if part of amino acids would cease participating in the folding/unfolding process) and as a result they will change the enthalpic component of the free energy of transition states. An analysis of the experimental results can lead to the same conclusions. *Consequently, the very first stage upon unfolding of GFP-*cycle3 *can indeed depend on the disruption of the mutual packing of amino acid residues on the protein surface.* The above conclusions on the effect of substitutions of single amino acid residues and ss-bridges allow creating the order of formation/destruction of structure elements upon folding/unfolding of GFP*-*cycle3 (Figure 8).

It should be added that such a sequence of GFP folding/unfolding is supported in other studies. The presence of the first stage, when the unfolding of the polypeptide chain of GFP*-*cycle3 does not occur but the mutual packing of the secondary structure elements is destroyed, is supported by NMR studies [22]. It was demonstrated that because of the presence of water molecules within the GFP-can at an early stage of melting of this protein its structure is “fractured” and this “fracture” differs from the usual unfolding of the polypeptide chain. Naturally it is not quite clear what conformation GFP*-*cycle3 acquires consequently. The experimental results show only that the changes occur in the total protein without unfolded regions (explanations are in [22]). The fact that beta-strands 1–3 (shown in red color in Fig. 8) either fold very rapidly or remain structured even in the denatured state is supported by several experimental methods, for example H/D exchange, NMR and far UV CD [6], [33]. There are also data verifying that the intermediate state of GFP*-*cycle3 is rather compact (I_2_ in Fig. 8), contains a large amount of the secondary structure but is highly mobile [6], [7]. The authors believe that this state can be described as the molten globule state.

### Conclusions

As expected, the basic result of this research is the experimentally determined order of folding/unfolding of secondary structure elements of GFP*-*cycle3 (Fig. 8). However, we believe that no less interesting is the fact how various mutations (substitutions of hydrophobic amino acids and ss-bridges) affected the free energy of GFP*-*cycle3. In particular, single amino acid substitutions had an effect mostly on the entropic component while ss-bridges on the enthalpic component of energy barriers. At first sight, this is somewhat surprising. When a hydrophobic amino acid is substituted (for example for alanine) it seems that the most substantial should be the change in the number of amino acid contacts, which in its turn implies a change in the enthalpic component of the free energy. On the contrary, upon incorporation of an ss-bridge it seems that we should influence the entropic component of the protein free energy. Nevertheless experimentally we obtained an exactly opposite result. Why? In our opinion, this can be explained in the following way. Single substitutions of hydrophobic amino acids influence mostly hydrophobic interactions rather than the contacts, i.e. they affect not the intensity of interactions but rather the search for required conformation. The matter is that hydrophobic interactions are entropic effects because they take place depending on expediency/non-expediency of hydrogen bond formation in the water-protein system. As a result it turns out that substitutions of hydrophobic amino acids with multiple contacts affect exclusively hydrophobic interactions and the effect of alteration of contacts has not been detected experimentally.

As for the ss-bridges, definitely they should change the entropic component of the free energy of mobile states, for example, the unfolded state or the molten-globule state, but hardly the entropy of well-packed states the main chain of which is not really mobile. We have studied just such states (native and close to it intermediate). For these states, it would be more essential for the ss-bridge to “freeze” the amino acid interactions in its vicinity and in this way influence the enthalpic component of energy barriers.

In case the above conclusions are more or less universal, it appears that the most “reliable” way to affect the free energy of tightly packed states (i.e. the protein stability) is to substitute hydrophobic acids. If it is required to change the transition states (i.e. the rate of different stages of folding/unfolding), it is necessary to incorporate ss-bridges.

### Some Recommendation that could be Helpful for Studying Multi-state Proteins

This research has allowed us not only make conclusions on the sequence of GFP*-*cycle3 folding, but also to elaborate some general recommendations helpful for studying the energy landscape of a multi-state protein. In particular how to choose amino acid residues for mutations and in this case what information on the protein could be expected.

1. Mutations of hydrophobic amino acids with multiple contacts in protein structure largely yield information on stable states of protein.
2. Incorporation of cross-links (disulfide bridges) gives information mainly on transition states or on mobile not tightly packed states like a molten globule state.
3. A single substitution of hydrophobic amino acids and incorporation of an ss-bridge affect the protein folding/unfolding pathway “from every corner”. A single substitution in the structure element that unfolds the last influences the stability of all protein states, at that in this structure element the ss-bridge would affect only the last stage of unfolding. In contrast, a single substitution in the structure element that unfolds the first would affect only the stability of the first (native) state, in this structure element the ss-bridge would change all the folding/unfolding stages of the protein.

Moreover another important conclusion suggests itself. Each of the mutations (single substitution of hydrophobic amino acids or incorporation of ss-bridges) taken separately does not allow us to analyze the pathway of folding of multistate proteins. It is necessary to use the above two types of mutations. This means that a multimutation analysis of multistate proteins is required. For short we have called this approach a μ-analysis.

What mutations should be chosen for the μ-analysis? The above considerations show that for single substitutions one should choose hydrophobic amino acids with a maximal possible amount of contacts. Such mutations should most of all affect the stable (tightly packed) states and to a lesser extent the transition states.

In contrast, for ss-bridges one should choose amino acids located on the protein surface not to destroy the hydrophobic contacts. Such mutations should most of all affect the transition states and to a lesser extent the tightly packed ones. In addition, ss-bridges should be designed so that they would “cross-link” the polypeptide chain inside the small structure element which is most likely formed at a folding stage. If different structure elements are “cross-linked”, we will have no necessary information on a certain structure element of the protein.

It is better to design a single substitution and an ss-bridge close to each other: this would provide an opportunity to analyze the effect of the two different mutations on the same structure element of the protein.

## Materials and Methods

### Protein Expression and Isolation

The GFP*-*cycle3 gene (pBAD-GFP-cycle3 produced by Maxygen) was transferred into the pET-28a vector («Novagen»). The resulting plasmid was designated pGFP*-*cycle3. Plasmids with the mutant GFP*-*cycle3 genes were constructed by a standard PCR technique using appropriate primers and a pET-28a vector as a template. For construction of the both single I14A, V112A, I161A, L201A and double V11CD36C, Q111CV93C, K162CQ184C, S202CT225C mutants, an appropriate mutation were introduce to pGFP*-*cycle3 by standard procedures using the Quick-Change kit («Stratagene», USA). The DNA sequences of all constructs were confirmed by the DNA sequence analysis.

GFP*-*cycle3 and it mutant forms were expressed and isolated as described elsewhere [8]. The purity of the isolated protein was checked by the SDS polyacrylamide gel electrophoresis. Since the ratio of the absorbance values at 395 and 280 nm is equal to 1.1–1.2 for pure GFP*-*cycle3, this 395/280 ratio is typically used as a GFP*-*cycle3 purity index [34]. The final sample prepared in this study had a 395/280 ratio of 1.1 or higher. The intensity of chromophore fluorescence in mutants was similar to that of GFP*-*cycle3 protein.

### Protein Chemistry

The protein concentration was determined by UV absorption at 280 nm with the extinction coefficient *A* ^0.1%^_280_ = 0.77 [8].

Disulfide bond formation was performed as follows. The pure protein was precipitated by 80% ammonium sulfate. The pellet was resuspended in 0.2 M Tris-HCl, pH 8.8, 0.2 M NaCl, 1 mM EDTA to a protein concentration of 3 mg/ml. The protein was oxidized by addition of oxidized and reduced glutathione to final concentrations of 10 mM and 2 mM, respectively. After 24 h incubation at room temperature, the glutathione was removed with a PD-10 Desalting column. Then quantity of free SH groups were defined by Ellmans reagent [35].

### Calorimetry

Calorimetric measurements were performed using a precision scanning microcalorimeter SCAL-1 (Scal Co. Ltd, Russia) with 0.33 ml glass cells, at a scanning rate of 0.25, 0.5, and 1.0 K/min and under the pressure of 2.5 atm [36]. The experiments were performed in 25 mM sodium phosphate buffer at pH 7.2. The protein concentrations in the experiment varied from 0.5 to 1.0 mg/ml. The experimental calorimetric profiles were corrected for the calorimetric baseline, and the molar partial heat capacity functions were calculated using a standard approach. The excess heat capacity (*C_p_^ex^*) was evaluated by subtraction of the linearly extrapolated initial and final heat capacity functions with correction for the difference of these functions by using a sigmoid baseline [37]. A characteristic value of the partial specific volume for typical globular proteins (0.73 cm^3^/g) was chosen arbitrarily, since it does not influence the calculated excess heat capacity.

### Estimation of the Rate Constants and Activation Energy from Melting Curves

The curves of the dependence of excess heat capacity versus temperature were approximated using the following equations.

The equation describing one-state irreversible denaturation (eq. 1). The excess heat capacity (*C_p_* ^ex^) describing this model is the following [38], [39]:
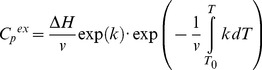
(4)where *T* and *T_0_* are the absolute temperature and the temperature of the beginning of the heat absorption curve, Δ*H* is the enthalpy change, *v = dT/dt* is the rate of temperature change of the sample in the calorimeter, and *k* is the denaration rate constant that can be expressed with the activation energy (*E^#^*) as follows:
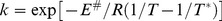
(5)where T* is the temperature parameter that is equal to the temperature at which the rate constant is 1 s−1.

The two-step models for the irreversible thermal denaturation.

The Lumry-Eyring model with the fast equilibrating first step (eq. 2), in which the rate of equilibrium establishing (*K*) for the first step is high in comparison with that (*k*) of the second step. The excess heat capacity (*C_p_* ^ex^) versus absolute temperature (*T*) is calculated from the equation [40]:
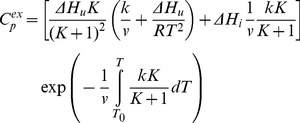
(6)where Δ*H_u_* and Δ*H_i_* are the change of enthalpy associated with the first and second steps, respectively. *v = dT/dt* is the scanning rate, *T* and *T_0_* are the absolute temperature and the temperature of the beginning of the heat absorption curve. *K* is the equilibrium constant for the first step that can be expressed as:



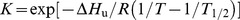
(7)
*k* is the rate constant for the second step that can be expressed as:
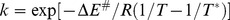
(8)where Δ*E* ^#^ is the energy of activation for the second process, *T*_1/2_ is the temperature at which *K* = 1, T* is the temperature parameter equal to the temperature at which the rate constant *k* is 1 min^−1^.

The other model of irreversible denaturation is a model involving two consecutive irreversible steps of denaturation (eq. 3). The equation for *C_p_* ^ex^ describing this model is the following [41]:
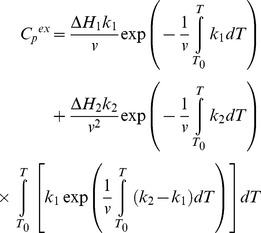
(9)where Δ*H_1_* and Δ*H_2_* are changes of enthalpy for the first and second steps, *k*_1_ and *k*_2_ are the rate constants for the first and second steps:



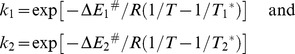
(10)On the basis of equations, the experimental profiles of GFP-cycle3 denaturation were fitted by the IgorPro program (WaveMetrics, Inc.).

### Simultaneous Fitting of Melting Curves

Fitting of the experimental curves was done simultaneously for three curves obtained at different heating rates. At that it was supposed that enthalpy values are individual parameters for each experimental curve, and activation energy values and temperature (T*, *T_1/2_*) are independent of the heating rate. For example, for a model involving two consecutive irreversible steps of denaturation (equations 3, 9, 10), parameters Δ*E_1_* ^#^, Δ*E_2_* ^#^, *T*_1_*, *T*_2_* were “fixed”, i.e. they should be the same for melting curves with heating rates 0.25, 0.5 and 1.0 K/min, whereas enthalpy values Δ*H*_1_ and Δ*H*_2_ were independent parameters. It was so because the protein melting enthalpy depends on the experimental conditions, i. e. the protein concentration, but the activation energy and T* are associated only with the curve shape (that depends only on transition state properties). Besides, such “fixing” of the parameters is an additional restriction that simplifies the choice of denaturation models. For example, it is seen from Fig. 2 that if such a melting curve of GFP*-*cycle3 is analyzed at the heating rate of 0.5 K/min, it can be concluded that the curve is well described with both two-state models (Fig. 2, B and C). However, only one model (3) can be used to simultaneously describe the three melting curves (at different heating rates) with the same activation energy values and T*.

We also tried to do another type of fitting when all parameters were individual for each melting curve at thee heating rates. When we used models 1 and 2 (equations 4 and 6), the activation energy values calculated from each melting curve were different for each one of the three curves. This was not the case when we used model 3. Though, this approach works well only upon the choice of the model. If the denaturation model is chosen, it is more expedient to do just the simultaneous curve fitting. It would lessen the error in calculating the activation energies and parameter T*. It should be also added that parameters Δ*E_1_* ^#^, Δ*E_2_* ^#^, *T*_1_*, and *T*_2_* determined upon fitting permit calculating the dependence of the logarithm of rate constant on reverse temperature (eq. 10).

### Experimental and Fitting Errors

Table 1 provides errors of the combined fitting and shows how well program IgorPro (WaveMetrics, Inc.) finds the only minimum among all the parameters (for three curves). The experimental error of the melting enthalpy determination is greater than the fitting error, being nearly equal to ±50 kJ/mol. However, the error of the determination of the rate constants is much lower. This is because of the fact that the Δ*E_1_* ^#^, Δ*E_2_* ^#^, T_1_*, and T_2_* parameters are associated only with the shape of the melting curves (equations 9 and 10). If the height of every melting peak is changed arbitrarily (which can occur, e.g. due to the error in the determination of protein concentration), this would affect the enthalpies (Δ*H_1_*, Δ*H_2_*) but would not affect the shape of the melting curves; i.e., the calculated activation energy values (Δ*E_1_* ^#^, Δ*E_2_* ^#^), temperature parameters (T_1_*, T_2_*), and rate constants (*k_1_*, *k_2_*). Furthermore, the calculation accuracy of the logarithm of rate constants is ±0.3 for ln(*k_1_*) and ±0.2 for ln(*k_2_*) and depends on the noisiness of the melting curves rather than on the height of melting peaks. The experimental error is about ±20 kJ/mol for the activation energy and ±1 K for the temperature parameters T*.

### Measuring the Kinetics of Heat Denaturation by the Method of Fluorescence

In measuring the kinetics of unfolding of GFP-cycle3, the temperature jump was achieved by a 100-fold dilution of cold solution of GFP-cycle3 (20 µl, 20°C) buffer heated to the temperature at which the kinetic experiment was performed (2 ml, 70–80°C). The final concentration of the protein in the cell was 0.1 mg/ml.

The kinetics of unfolding was measured by flurescence at the wavelength of the fluorescence of the GFP-cycle3 (excitation 400 nm, emission 510 nm). The measurements were made with a Shimadzu RF-5301 PC spectrofluorimeter (Japan). The curves of dependence of the fluorescence intensity versus time were approximated by the following equation:
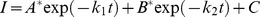
(11)where *I* is the fluorescence intensity; *t* is the time; *A, B, C, k_1_, k_2_*, are approximation parameters.
